# Natural Magnetite: an efficient catalyst for the degradation of organic contaminant

**DOI:** 10.1038/srep10139

**Published:** 2015-05-11

**Authors:** Hongping HE, Yuanhong ZHONG, Xiaoliang LIANG, Wei TAN, Jianxi ZHU, Christina Yan WANG

**Affiliations:** 1 CAS Key Laboratory of Mineralogy and Metallogeny, Guangzhou Institute of Geochemistry, Chinese Academy of Sciences, Guangzhou 510640, China; 2 University of Chinese Academy of Sciences, Beijing 100049, China; 3 Guangdong Provincial Key Laboratory of Mineral Physics and Materials, Guangzhou 510640, China

## Abstract

Iron (hydr)oxides are ubiquitous earth materials that have high adsorption capacities for toxic elements and degradation ability towards organic contaminants. Many studies have investigated the reactivity of synthetic magnetite, while little is known about natural magnetite. Here, we first report the reactivity of natural magnetites with a variety of elemental impurities for catalyzing the decomposition of H_2_O_2_ to produce hydroxyl free radicals (^•^OH) and the consequent degradation of *p*-nitrophenol (p-NP). We observed that these natural magnetites show higher catalytic performance than that of the synthetic pure magnetite. The catalytic ability of natural magnetite with high phase purity depends on the surface site density while that for the magnetites with exsolutions relies on the mineralogical nature of the exsolved phases. The pleonaste exsolution can promote the generation of ^•^OH and the consequent degradation of p-NP; the ilmenite exsolution has little effect on the decomposition of H_2_O_2_, but can increase the adsorption of p-NP on magnetite. Our results imply that natural magnetite is an efficient catalyst for the degradation of organic contaminants in nature.

By mass, iron is the fourth most abundant element in the earth’s crust, being found in the form of 16 iron (hydr)oxides. These Fe-rich minerals affect the behavior and fate of environmental pollutants, notably heavy metals and organic contaminants^1^,^2^. The capacity of natural iron (hydr)oxides for adsorbing and degrading such pollutants depends on the composition, structure, and property of the minerals^3^,^4^.

Unlike other iron (hydr)oxides, magnetite uniquely contains both Fe^2+^ and Fe^3+^ in its structure. The formula of magnetite can be written as AB_2_O_4_ where A denotes the tetrahedral site and B denotes the octahedral site. All Fe^2+^ and half of Fe^3+^ occupy 16 of 32 available octahedral sites in the unit cell, while the rest of Fe^3+^ occupy 8 of 64 available tetrahedral sites in magnetite^5^. Magnetite is a semiconductor that can initialize oxidation/reduction reactions, and its inverse spinel structure makes it a stable phase in various geochemical processes^6^,^7^, resulting in a wide distribution in various earth surface environments^8^.

Among the magnetites with geological geneses, magmatic magnetite is of great importance in terms of abundance, distribution and application. The crust of the earth consists of rocks, which are the primary sources of magnetite and supply most of iron cycled through the earth’s surface ecosystems. Among the three basic groups of rocks (i.e., magmatic, metamorphic and sedimentary), magmatic rocks (volcanic or extrusive and plutonic or intrusive) cover most part of the solid earth surface, in which the major Fe oxides are magnetite and ilmenite, and to a lesser extent, hematite^9^,^10^. Different from biogenic magnetite and neoformed magnetite in sediment and soil, magmatic magnetite has extensive isomorphous substitution (e.g., Ti^4+^ and V^3+^ for Fe^3+^ ) in its structure^11^. Previous studies about synthetic magnetites have demonstrated that the incorporation of transition metals greatly affects the catalytic activity of synthetic magnetite. For example, incorporation of Mn^12^, Co^12^, V^13^, and Cr^14^ enhances the heterogeneous catalytic activity of magnetite in the degradation of organic pollutants by hydrogen peroxide (H_2_O_2_), while that of Ni has an inhibitory effect^12^.

Magmatic magnetite can also form a complete solid solution with magnesioferrite (MgFe_2_O_4_) and ulvöspinel (Fe_2_TiO_4_), and partial solid solutions with franklinite (ZnFe_2_O_4_), jacobsite (MnFe_2_O_4_) and trevorite (NiFe_2_O_4_)^15^. In magmatic magnetite, various micro-intergrowth textures have been observed such as sandwich-type and trellis-type ilmenite lamella in the magnetite matrix^16^, which are remarkably different from those synthetic ones, and may exert great effects on the reactivity of magnetite towards organic contaminants.

Like the synthetic magnetite, natural magnetite can potentially serve as a heterogeneous catalyst for the degradation of various organic compounds through the heterogeneous catalytic reaction since H_2_O_2_ is usually present in rain water and soil^17^. In this respect, the reactivity of magnetite may be enhanced by either promoting the generation of hydroxyl radicals from H_2_O_2_, or increasing the adsorption capacity for the organic contaminants. This observation applies to synthetic magnetite that forms under mild experimental conditions and has a homogeneous chemical composition and structure^18^. It is unknown whether natural magnetite of magmatic or metamorphic origins with variable chemical compositions and microstructures can be active in catalyzing organic decomposition, which is, however, of high importance for well understanding the reactivity of natural magnetite.

In seeking an answer to this question, we have tested natural magnetite samples from Fe-Ti oxide-bearing, mafic-ultramafic intrusions, anorthosite massif, hydrothermal iron oxide deposit and metamorphosed banded iron formation from northeastern China (Table 1 and Fig. S1). The samples collected from Tianshan, Zankan, Hannan, Damiao and Panzhihua Districts, were labeled as TS, ZK, HN, DM and PZH, respectively. The chemical composition and texture of the samples were characterized using X-ray diffraction (XRD), electron probe microanalysis (EPMA), inductively coupled plasma atomic emission spectroscopy (ICP-AES), scanning electron microscope (SEM) with backscattering electron (BSE) imaging, X-ray absorption fine structure (XAFS) spectroscopy as well as BET specific surface area and surface site density measurements. The capacity of these magnetite samples in generating hydroxyl radicals (^•^OH), and their capacity in catalyzing the degradation of *p*-nitrophenol (p-NP) were assessed by batch experiments. We chose p-NP as the model organic contaminant, because p-NP is one type of hazardous waste that is mainly produced during chemical processes, including petrochemical manufacturing, oil refining, rubber, wood preservation operations, pulp and paper mills as well as in the production of pesticides, paints and plastics. p-NP is highly persistent, bioaccumulative and toxic, and listed as priority pollutant by US Environmental Protection Agency^19^,^20^.

## Results and discussion

### Mineralogical characterization

**Figure 1:**
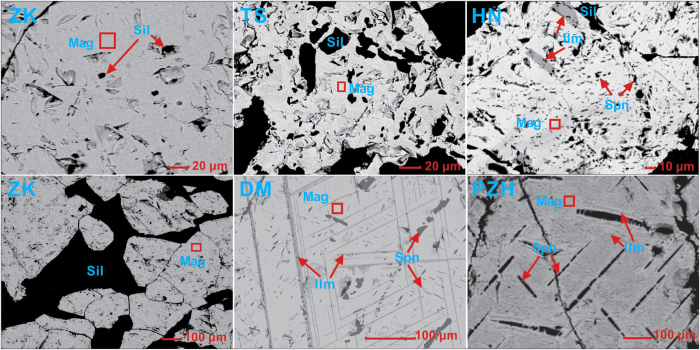
Backscattered electron (BSE) images of natural magnetite samples (Mag = magnetite; Sil = silicate; Ilm = ilmenite; Spn = spinel).

The BSE images (Fig. 1) show that TS and ZK are composed of relatively pure magnetite without any exsolution while HN contains a small amount of granules or lamellae of ilmenite. DM contains exsolved thick sandwich-type ilmenite lamellae and trellis-type pleonaste-ilmenite lamellae, whereas PZH contains trellis-type pleonaste lamellae and ultrafine cloth-type ilmenite lamellae. The presence of these exsolved mineral phases is also indicated by the corresponding micro-Raman spectra and micro-XRD patterns^21^.

**Figure 2:**
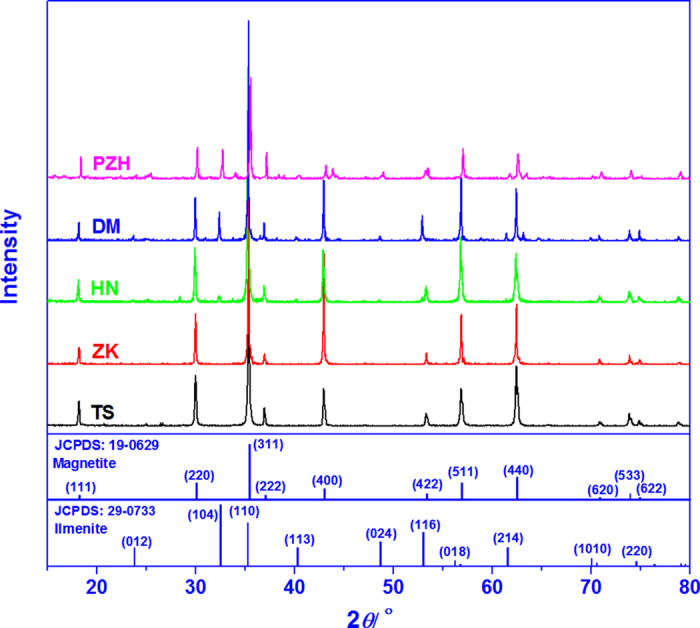
XRD patterns of the five magnetite samples, a standard magnetite (JCPDS:19-0629), and a standard ilmenite (JCPDS:29-0733).

The powder XRD patterns of HN, DM and PZH (Fig. 2) indicate the presence of both magnetite and ilmenite. Magnetite is the only phase in TS and ZK as indicated by their XRD patterns. Because of structural similarity, pleonaste could not be distinguished from magnetite by XRD when they coexist in the samples. To determine the ratio of exsolved mineral to magnetite matrix, a combination of Rietveld and Reference Intensity Ratio (RIR) methods was applied to calculate mineral components based on the XRD patterns^22^,^23^ (Table 2). In DM and PZH, the spinel phase including magnetite and pleonaste is dominant in the samples which also contain a significant amount of ilmenite. Apparently, the Fe-containing exsolved phases (ilmenite or pleonaste) are difficult to separate from the magnetite matrix during purification because of their textural intergrowths.

Other factors affecting the activity of magnetite in catalyzing organic degradation are chemical constitution and metal coordination. As indicated by ICP-AES measurements (Table 3), the total Fe content for TS, ZK, and HN is very close to the theoretical value for stoichiometry of Fe_3_O_4_ (72.36 *wt*%) whereas those for DM and PZH are considerably lower. All five samples contain trace amounts of various transition metals (Cr, Mn, Co, Ni). The chemical composition of the host matrices and exsolved phases was also measured by EPMA (Table S1). In the absence of exsolution, the magnetite matrix of TS and ZK is composed of Fe and O with a low content of trace elements (Fig. S2). Likewise, the matrix of DM and HN with exsolution, is made of magnetite. The content in Fe and Ti of the exsolved ilmenite in DM and HN is close to the standard stoichiometry of ilmenite. The high content of Ti, relative to that of Al and Mg (Table 3), indicates that the exsolved phase in DM and HN is majorly ilmenite, with only a small contribution from pleonaste. Most of Ti in PZH is associated with magnetite matrix (Table S1). The RIR analysis gave an ilmenite content of ~38.1%, suggesting that the PZH matrix is composed of magnetite and ilmenite. Further, the exsolved phase of in PZH has a high content of Al, Mg and Fe, and a low content of Ti, indicative of pleonaste with a minor amount of ilmenite.

The coordination environment of Fe and Ti was investigated by K edge XAFS. The absorption edge positions of Fe species in TS and ZK are close to that in synthetic magnetite (Fig. S3.a). For DM, HN and PZH, they are even closer to that of Fe^2+^ in FeO and ilmenite. The slight shift to lower energy is probably due to the contribution of Fe^2+^ in ilmenite. The filtered EXAFS oscillations, *k*^3^χ(*k*), TS and ZK are almost identical with those of the synthetic mineral, and clearly different from those of ilmenite (Fig. S3.b). In line with the RIR analysis results, this observation indicates that the local environment of Fe cations in the TS and ZK is similar to that in the synthetic analogue. But for DM, HN and PZH, the oscillations display slight shift to high k value, especially in range of 7–9.5 Å^−1^, and their positions become close to those of ilmenite, suggesting that partial Fe^2+^ in these samples is incorporated in the ilmenite structure, consistent with the corresponding XRD and BSE observations (Figs. 1 and 2).

In order to assess whether Ti cation is on the lattice of magnetite rather than ilmenite, the Ti K-edge XAFS spectra of the natural magnetites were measured (Fig. S4). Because of their relatively high Ti content (Table 3), only DM and PZH showed the Ti K-edge signal. The absorption position of Ti for DM and for PZH is close to that of Ti^4+^ in rutile, ilmenite, and titanomagnetite, indicating that the Ti species in the DM and PZH is Ti^4+^ (Fig. S4.a). However, The filtered EXAFS oscillations for natural magnetites are similar to that for ilmenite, but clearly different from those for titanomagnetite (Fig. S4.b), indicating that Ti in DM and PZH is incorporated in ilmenite rather than being substituted in the magnetite structure.

The Mössbauer hyperfine parameters (Fig. S5, Table S2) also confirm the matrix of TS and ZK samples is magnetite and most of the trace cations are not in the structure of magnetite, which is consistent with the EDS analysis results. In HN sample, Fe^2+^ in magnetite was oxidized without changing the spinel structure, during its formation in the subduction-related environment with high oxygen fugacity^24^. In DM sample, the trace metal cations, e.g., V, Cr and Mn, preferentially occupy the tetrahedral sites of magnetite, while in PZH sample, trace metal cations do not enter the structure of magnetite. The detailed discussion of Mössbauer spectra results has been presented in Text S1.

Table 4 lists the lattice parameter (*a*_0_), BET specific surface area, *pH*_zpc_, and surface site density of the five samples. The surface site density (*D*_s_) of the samples, is widely variable, with the highest value (147.72 sites nm^−2^) for TS and a decreasing in the order DM > PZH > HN > ZK, with the lowest value (0.26 sites nm^−2^) for ZK.

### Natural magnetite catalyzed hydroxyl radical (^•^OH) generation

The iron oxide-catalyzed degradation of organic pollutants in the presence of H_2_O_2_ is mediated by hydroxyl radicals (^•^OH), generated during the reaction between H_2_O_2_ and the mineral (Eqs. (1),(2))^25^. The ^•^OH radical has a high redox potential (2.73 V) that can oxidize most organic molecules^26^.


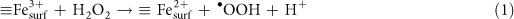



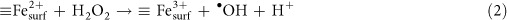


**Figure 3:**
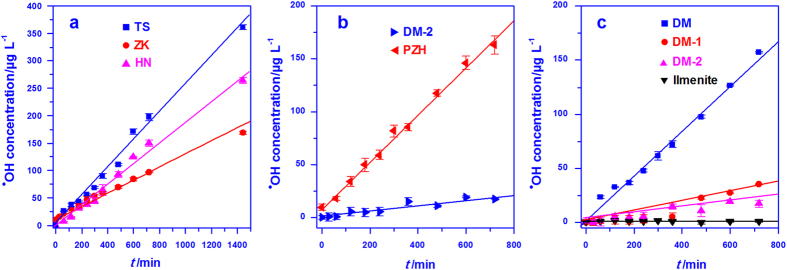
Kinetics of ^•^OH generation from hydrogen peroxide catalyzed by natural magnetite samples and ilmenite at neutral pH. (**a**) pure magnetites; (**b**) effect of exsolved phase; and (**c**) effect of exsolved ilmenite. The concentration of mineral was 1.0 g L^−1^, and that of H_2_O_2_ was 10 mmol L^-1^.

The generation of ^•^OH at neutral pH catalyzed by TS, ZK, and HN, increased linearly with reaction time (Fig. 3a), obeying zero-order kinetics (Eq. (3), correlation coefficient *R*^2^ > 0.99),


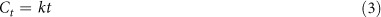


where *C*_*t*_ denotes ^•^OH concentration (μg L^−1^), *t* is the reaction time (min), and *k* is the reaction rate constant (μg L^−1^ min^−1^).

The *k* values for TS, HN, and ZK were 0.24, 0.19, and 0.12 μg L^−1^ min^−1^, respectively, showing that the catalytic activity decreases in the order TS > HN > ZK. Although both TS and ZK magnetites are highly pure (no exsolved ilmenite or pleonaste), the catalytic activity of TS was twice as high as that of ZK. Even the HN sample with 7 wt% ilmenite was superior to ZK in terms of catalytic performance. Therefore, the compositional purity is not the unique factor controlling the reactivity of natural magnetites in ^•^OH generation.

All five magnetite samples have a similar pH_zpc_, a small BET surface area, but a markedly different surface site density (Table 4). Interestingly, the *D*_*s*_ values for the relatively pure TS, HN, and ZK are positively correlated with the rate constant (*k*), suggesting that surface site density has a profound influence on the reactivity. The reactivity of iron (hydr)oxides is closely related to the surface hydroxyl groups exposed on particle surfaces^27^. In aqueous media, these groups can acquire or lose protons, depending on suspension pH values^28^. Acidic conditions (pH < *pK*_a1_) promotes protonation of surface hydroxyls (Eq.(4)), yielding ≡Fe(II,III)OH_2_^+^ as the dominant surface species. Close to the pH_pzc_, the ≡Fe(II,III)OH species becomes dominant, while under alkaline conditions (pH > *pK*_a2_), the surface hydroxyl are deprotonated (Eq.(5)), yielding ≡Fe(II, III)O^–^ as the dominant species^29^. As all five magnetite samples have similar *pH*_zpc_ values ranging from 6.7 to 7.0, under the experimental conditions used in this study, ≡Fe(II,III)OH would be dominant. Previous study^30^ shows that, the presence of ≡Fe(II,III)OH surface hydroxyls was found to promote the adsorption of H_2_O_2_, and conversion of H_2_O_2_ into ^•^OH rather than into H_2_O and oxygen. Therefore, with the increase of surface site density, which is positively related to the hydroxyl group on magnetite surface, natural magnetite displayed higher activity in ^•^OH generation.


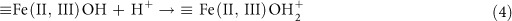



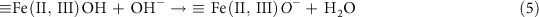


The effect of exsolved phases on catalysis was also investigated using a series of DM magnetite with different contents (wt%) of ilmenite but similar surface site density (30–40 sites nm^−2^). These samples, denoted as DM (21.5%), DM-1 (27.2%), and DM-2 (37.4%), were obtained by gravity and magnetic separation (Tables 2 and 3). In order to differentiate the contribution of exsolved pleonaste to catalytic activity from that of ilmenite, we compared the ^•^OH generating capacity of PZH with that of DM-2. Both the two samples (PZH and DM-2) contain similar amounts of Fe and Ti, and close content of ilmenite, but the exsolved phase in PZH is mainly composed of pleonaste (with a small amount of ilmenite), while the reversed situation applies to DM-2. The generation of ^•^OH, catalyzed by both PZH and DM-2, followed zero-order kinetics (Fig. 3b, *R*^2^ > 0.95). The rate constant (*k*) for PZH (0.22 μg L^−1^ min^−1^), however, was about eight times larger than that for DM-2 (0.027 μg L^−1^ min^−1^), indicating that the exsolved phase of pleonaste greatly improved the catalytic ability of natural magnetites in ^•^OH generation.

The positive effect of exsolved pleonaste on the heterogeneous catalytic ability of natural magnetite may be ascribed to its spinel structure, which is consistent with the strong catalytic ability of the other spinel structures, such as chromite^31^ and zinc spinel^32^. This observation may be explained in terms of the feasible accommodation into octahedral sites of transition metal cations (e.g., Fe, Mn, Cr and V) (Table S1), which can be reversibly oxidized and reduced, and accelerate the electron transfer within the structure. Therefore, the presence of exsolved pleonaste improved the reactivity of natural magnetite.

The effect of exsolved ilmenite on the reactivity of magnetite was investigated by measuring ^•^OH generation rate for DM, DM-1, and DM-2 under identical experimental conditions. The measurement results show that the rate of ^•^OH generation decreased with an increase of ilmenite exsolution content in the samples (Fig. 3c), giving a kinetic constant of 0.21, 0.049 and 0.027 μg L^−1^ min^−1^ for DM, DM-1, and DM-2, respectively (*R*^2^ > 0.95). Thus, the ^•^OH generating efficiency decreased in the order DM > DM-1 > DM-2, with DM producing three times more ^•^OH over 12 h than DM-2. Even for the natural ilmenite, no ^•^OH was generated during the tested process.

The catalytic efficiency of minerals in decomposing organic pollutants is directly correlated with catalyst dosage and H_2_O_2_ concentration^33^, which were investigated in ^•^OH generation with the presence of TS magnetite. A gradual increase in the yielding of ^•^OH was detected when the magnetite dosage increased from 0 to 1.5 g L^−1^ (Fig. S6.a). The processes followed the zero-order kinetics (Table S3). The ^•^OH production also increased with H_2_O_2_ concentration (Fig. S6.b), indicating that hydrogen peroxide is the source of hydroxyl radicals. Up to a concentration of 10 mmol L^-1^, ^•^OH production increased linearly with the reaction time. When the peroxide concentration was further increased, the dynamics of ^•^OH generation changed from being linear to a power function (Table S3). The generation rate was obviously enhanced with the increase of H_2_O_2_ concentration. From previous study^34^, the increase of H_2_O_2_ concentration would increase the dissolved iron concentration and induce the homogeneous catalytic oxidation. It may be the main reason for the fast ^•^OH generation at high H_2_O_2_ concentration.

In this study, we mostly used a low catalyst dosage (1.0 g L^−1^) and H_2_O_2_ concentration (10 mmol L^−1^) in order to minimize interference from the homogeneous reaction catalyzed by leaching iron. It is noteworthy that even when the H_2_O_2_ concentration is as low as 1 × 10^−3^ mmol L^−1^, the oxidation of Fe(II) by H_2_O_2_ is about 700 times faster than that by O_2_^35^. Thus, although H_2_O_2_ concentration in soil and sediment is typically 100−1000 times lower than that used here^17^, the results obtained may well be valid in the natural environment.

### The degradation of *p*-Nitrophenol

**Figure 4:**
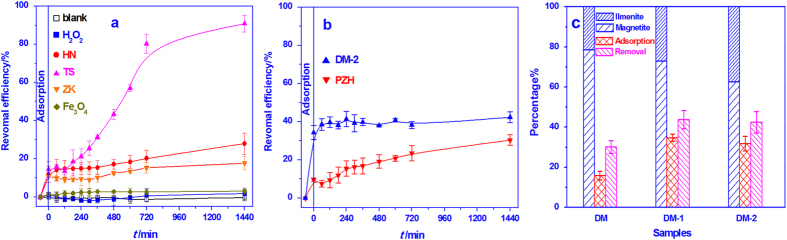
Heterogeneous catalytic degradation of *p*-nitrophenol by magnetites in the presence of H_2_O_2_ at neutral pH. (**a**) pure magnetites; (**b**) effect of exsolved phase; and (**c**) effect of exsolved ilmenite. Catalyst: 1.0 g L^−1^; H_2_O_2_: 10 mmol L^-1^; p-NP: 10 mg L^−1^.

The catalytic activity of the natural magnetite samples was further evaluated by following the degradation of *p*-nitrophenol (p-NP) in the presence of H_2_O_2_ at neutral pH. Little, if any, degradation occurred in the system containing only H_2_O_2_ (Fig. 4a), indicating that peroxide alone was not capable of oxidizing p-NP. When only magnetite was present, the removal of p-NP was due to adsorption by the mineral. Appreciable degradation of p-NP, however, was observed in the system containing both H_2_O_2_ and magnetite. For the pure magnetite samples (TS, HN and ZK), the p-NP removal by adsorption was close to 10%, while the efficiency in p-NP degradation was significantly different. Within a 24 h period, about 95.0, 27.7, and 17.8% of p-NP was degraded by TS, HN, and ZK, respectively. The order of decreasing efficiency, TS > HN > ZK, is the same as that of ^•^OH generating capacity. This indicates that p-NP degradation was mediated by hydroxyl radicals.

As shown by Fig. 4b, before the addition of H_2_O_2_, the adsorption efficiency of p-NP was about 9.7% by PZH and 34.3% by DM-2. Therefore, DM-2 was more efficient than PZH in removing p-NP by adsorption, although the rate of ^•^OH generation by DM-2 was much lower than that by PZH. PZH, however, showed high and sustained degradation efficiency throughout the period of measurement. Although PZH could remove 30.4% p-NP within 24 h as compared with 42.3% by DM-2, the degradation efficiency of PZH (20.7%), which was obtained by deducting the adsorption efficiency from the removal efficiency, was about 2.5 times higher than that of DM-2 (8.0%). Again, this observation is consistent with the difference in the ^•^OH generation rate between these two magnetite samples. Further, the presence of exsolved ilmenite in DM-2 appeared to promote p-NP adsorption, while that of exsolved pleonaste in PZH tended to accelerate p-NP degradation. The capacity of ilmenite for adsorbing p-NP was further evaluated by comparing the efficiency of DM, DM-1, and DM-2 in degrading p-NP. The p-NP adsorption increases with the increase of exsolved ilmenite (Fig. 4c), confirming that the presence of ilmenite enhances adsorption capacity.

For comparison, the reactivity of synthetic pure magnetite (Fe_3_O_4_) was also tested for p-NP degradation. In the presence of nano-sized magnetite (Fe_3_O_4_) and H_2_O_2_, no obvious degradation was observed during whole process (Fig. 4a), illustrating the higher catalytic performance of natural magnetite than the synthetic pure magnetite did.

The catalyzed degradation of p-NP by TS magnetite was monitored by UV-Vis absorption spectroscopy. The spectrum of the blank system showed two peaks at ~227 and 317 nm (Fig. S7), corresponding to phenyl ring and π-conjugation between the phenyl ring and nitro group, respectively^36^. In the presence of magnetite and H_2_O_2_, p-NP was progressively removed as indicated by the gradual diminution of the 317 nm peak, and its disappearance after 1440 min due to p-NP degradation by hydroxyl radicals^37^. This observation clearly demonstrates that natural magnetite is capable of catalyzing the decomposition of p-NP in the presence of H_2_O_2_. In all instances, the concentration of dissolved Fe cations was below the limit of detection, indicating the heterogeneous character of the ^•^OH generation and p-NP degradation processes.

**Figure 5:**
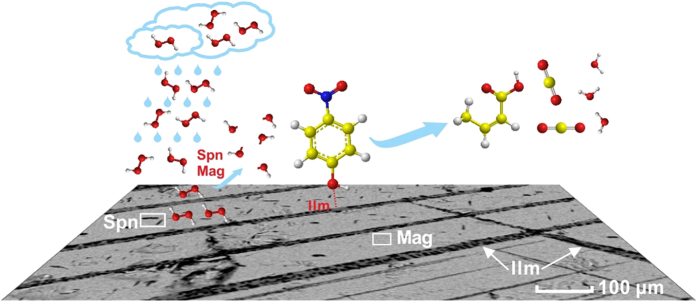
The schematics of magnetite-catalyzed degradation processes of *p*-nitrophenol in the presence of H_2_O_2_ (Mag = magnetite; Ilm = ilmenite; Spn = spinel).

The present study is the first to demonstrate that natural magnetite can generate hydroxyl radicals (^•^OH) from H_2_O_2_, and effectively catalyze the degradation of p-NP (Fig. 5). Surface site density appears to be an important factor affecting the catalytic activity of natural magnetite. The presence of exsolved spinel (e.g., pleonaste) tends to promote catalytic activity owing to the incorporation of transition metals with variable valence. On the other hand, the presence of exsolved ilmenite enhances the capacity of magnetite for adsorbing p-NP, but has little effect on its ability to generate ^•^OH from H_2_O_2_.

Natural magnetites have a complex chemical composition, especially with respect to the site and extent of isomorphous substitution (of transition metal cations for iron) within the structure. For this reason, the surface properties of these minerals need to be assessed using a variety of advanced instrumental techniques. Another source of complexity is the frequent presence of exsolved phases in natural magnetites. As such, the catalytic reactivity of these minerals is much more complicated than that shown by synthetic magnetite. Although the present study is less than comprehensive, the results provide valuable insight into the factors affecting the reactivity of natural magnetites, and the mechanisms underlying their capacity for catalyzing the decomposition of organic pollutants through a heterogeneous process.

## Methods

### Sample preparation

Magnetite particles were separated from crushed and ground ores by passing through a 200-mesh sieve (0.075 mm). Magnetite was hand-picked under a binocular, followed by magnetic and gravity separation so as to obtain highly pure materials. The separated magnetite particles were used for structural characterization and degradation experiments. The backscattered electron (BSE) images were obtained using thin sections of the parent rocks. Synthetic pure magnetite prepared through a precipitation-oxidation method have been characterized in the previous study^38^.

### Sample characterization

The BSE images were obtained from a JEOL JXA-8100 Superprobe with an accelerating voltage of 15 kV, a beam current of 20 nA, a spot diameter of 1−2 μm, and a peak counting time of 10 s. Data reduction was carried out using ZAF correction. The analysis of major elements was calibrated against multiple silicate and pure oxide standards obtained from SPI Supplies, Inc., USA. The chemical composition was determined using a Varian Vista-PRO inductively coupled plasma atomic emission spectroscopy (ICP-AES), with an analytical uncertainty of 1−2%. The digestion procedure before ICP-AES has been presented in Text S2. The XRD patterns were recorded between 10 and 80° (2*θ*) at a scanning rate of 1° min^−1^ using a Bruker D8 advance diffractometer with Cu *Kα* radiation (40 kV and 40 mA). The K-edge XAFS spectra were obtained using the 1W1B beamline at the Beijing Synchrotron Radiation Facility (BSRF). The storage ring operating conditions were 2.2 GeV electron energy and 250 mA beam current. The 1W1B is a focused X-ray beamline, obtained from a Si(111) double crystal monochromator. The beam size at the sample position was about 900 × 300 μm^2^. The K-edge spectra were acquired at room temperature in transmission mode. X-ray 3*d* foil sets were used to perform energy calibration of the monochromator for target elements. The sample thickness was optimized to give an edge jump of 0.6-1, depending on the content of target element. The IFEFFIT software was used for data analysis. The ^57^Fe Mössbauer spectra were collected at room temperature with a 25 mCi ^57^Co/Pb source in transmission mode with a constant acceleration. Calibration of velocity scale was performed with reference to 25 μm thick α-Fe at room temperature. Lorentzian doublets were used for fitting the areas of sub-spectra. The BET specific surface area of the samples was measured by physisorption of N_2_ at 77 K to samples that had been degassed at 433 K for 12 h, using an ASAP 2020 instrument. The surface site density (*D*_s_) of magnetite particles, i.e., the number of proton-active sites (per nm^2^), was determined by acid-base potentiometric titration. The detailed procedure has been presented in Text S3. The intrinsic acidity constants of the surface (p*K*_a1_ and p*K*_a2_), and the zero point of charge (*pH*_zpc_), were obtained from the Gran titration curves (Fig. S8).

### Hydroxyl radical production

The generation of ^•^OH was traced during the catalytic decomposition of H_2_O_2_ by the natural magnetite samples. The initial solution, containing magnetite particles (1.0 g L^−1^) and dimethyl sulfoxide (250 mmol L^−1^), was stirred for 1 h to ensure particle dispersion. Then 10 mmol L^−1^ H_2_O_2_ was added to initiate ^•^OH generation. The initial pH was adjusted to 7.0 by dropwise addition of H_2_SO_4_ (0.05 mol L^−1^) and NaOH (0.1 mol L^−1^). At given intervals, 2.0 mL of the suspension was passed through a 0.22 μm nylon filter to separate the particles. Then 1.0 mL of filtrate was placed in a 2.0 mL vial, and mixed with 0.1 mL of a 2,4-dinitrophenylhydrazine (DNPH) solution (0.5 mmol L^−1^) to form 2,4-dinitrophenylhydrazones (DNPHo). The mixture was analyzed by high performance liquid chromatography (HPLC, Shimadzu LC-20A), equipped with an Inertisil ODS-SP column (150 mm × 4.6 mm, 5 μm particles). The mobile phase used was a mixture of methanol and water (60/40 v/v), and the detector UV wavelength was set at 355 nm. The retention time was about 4.08 min for DNPH and 7.77 min for DNPHo. Details of the mechanisms involved in the detection of ^•^OH radicals have been given by Oliva-Teles et al.^39^.

### The degradation of *p*-Nitrophenol

The degradation of *p*-nitrophenol (p-NP) was performed in a conical flask, containing a 250 mL suspension of magnetite particles (1.0 g L^−1^) and p-NP (10 mg L^−1^). After adjusting the initial pH to 7.0, using H_2_SO_4_ (0.05 mol L^−1^) and NaOH (0.1 mol L^−1^), the suspension was stirred for 1.0 h, the predetermined time for achieving adsorption equilibrium. Then H_2_O_2_ (10 mmol L^−1^) was added to initiate p-NP degradation. At given intervals, a 2.0 mL aliquot was taken out and immediately diluted to 5.0 mL, and analyzed for p-NP concentration by UV-Vis spectroscopy (Perkin Elmer Lambda 850) at λ_max_ = 318 nm. The UV-Vis spectral changes were monitored in order to assess the degradation products. The concentration of leached Fe cations was determined using a Perkin Elmer-3100 Flame Atomic Absorption Spectrophotometer (FAAS) with the hollow-cathode lamps operating at a wavelength of 248.3 nm.

## Author Contributions

H. P. H. and Y. H. Z. conceived and designed the experiments. Y. H. Z. and X. L. L. analyzed the data. H. P. H., X. L. L. and Y. H. Z. wrote the manuscript. W. T., C. Y. W. and J. X. Z. were involved in the related discussion and helped to improve the quality of the manuscript.

## Additional Information

**How to cite this article**: He, H. P. *et al*. Natural Magnetite: an efficient catalyst for the degradation of organic contaminant. *Sci. Rep*. **5**, 10139; doi: 10.1038/srep10139 (2015).

## Supplementary Material

Supplementary Information

## Figures and Tables

**Table 1:** Occurrence, host rock, ore type, morphology, and exsolved phase texture for the magnetite samples.

Sample	Occurrence	Host rock	Ore type	Morphology
TS	Dahalajunshan formation, South Tianshan Orogenic Belt, SW China	Andesite	Massive	Euhedral/subhedral
ZK	Bulunkuole formation, Sanjiang orogenic belt, SW China	Banded iron formation in chert	Banded structure/massive/disseminated	Euhedral/subhedral/granoblastic
HN	Bijigou mafic-ultramafic intrusion in the Hannan complex, Qinling Orogenic Belt, Central China	Gabbro	Net-texture/disseminated	Subhedral/anhedral
DM	Damiao anorthosite massif, North China Craton	Anorthosite	Massive	Coarse-grained subhedral/ anhedral
PZH	Panzhihua layered intrusion in the Emeishan large igneous province, SW China	Gabbro	Massive	Euhedral

**Table 2:** Mineral phase contents of the samples obtained by RIR method.

Samples	*ω*_spinel_*	*ω*_ilmenite_
TS	100.0	0
ZK	100.0	0
HN	93.0	7.0
PZH	61.9	38.1
DM	75.8	24.2
DM-1	72.8	27.2
DM-2	62.6	37.4

*ω* denotes percentage phase content. *ω*_spinel_* includes both magnetite and pleonaste.

**Table 3:** Chemical compositions of the magnetite samples measured by ICP-AES (*wt*%).

Samples	Fe	Ti	V	Cr	Mn	Co	Ni	Al	Mg	O*
TS	71.93	0.12	0.11	0.02	0.06	0.00	0.01	0.26	0.56	26.94
ZK	71.82	0.11	0.11	0.02	0.07	0.01	0.01	0.50	0.50	26.85
HN	71.36	1.97	0.73	0.02	0.09	0.01	0.00	1.19	0.44	24.20
PZH	58.67	9.11	0.32	0.02	0.32	0.02	0.00	2.39	2.23	26.92
DM	66.52	4.90	0.41	0.19	0.13	0.01	0.02	2.15	0.74	24.92
DM-1	65.71	5.18	0.39	0.18	0.14	0.01	0.02	1.92	0.80	25.63
DM-2	60.30	8.56	0.45	0.18	0.15	0.01	0.02	1.99	0.95	27.37

O weight content* is the calculated value by deducting the weight contents of analyzed metals from 100%.

**Table 4:** Lattice parameter (*a*_0_), BET specific surface area, *pH*_zpc_, and surface site density (*D*_s_) of the natural magnetite samples.

Samples	*a*_0_(Å)	BET area (m^2^ g^−1^)	*D*_*s*_ (sites nm^−2^)	*pK*_a1_	*pK*_a1_	pH_zpc_
TS	8.410	0.70	147.72	3.97	10.14	7.06
ZK	8.402	3.30	0.26	3.82	10.06	6.94
HN	8.420	0.54	7.46	3.70	9.88	6.79
DM	8.404	2.46	34.33	3.79	9.81	6.80
PZH	8.407	1.41	32.95	3.86	9.83	6.71
