# Supplementary material for: Natural Magnetite: an efficient catalyst for the degradation of organic contaminant
Source: Sci Rep. 2015 May 11;5:10139. doi: 10.1038/srep10139 (PMC4426601; doi:10.1038/srep10139)
Supplement: Supplementary Information [file srep10139-s1.pdf]

Supplementary information

Natural Magnetite: an efficient catalyst for the degradation of organic contaminant

Hongping HE[1,3*], Yuanhong ZHONG[1,2,3], Xiaoliang LIANG[1,3], Wei TAN[1,2,3], Jianxi ZHU[1,3], Christina Yan WANG[1,3]

[1] CAS Key Laboratory of Mineralogy and Metallogeny, Guangzhou Institute of Geochemistry, Chinese Academy of Sciences, Guangzhou 510640, China

[2] University of Chinese Academy of Sciences, Beijing 100049, China

[3] Guangdong Provincial Key Laboratory of Mineral Physics and Materials, Guangzhou 510640, China

[*] Corresponding author: Hongping HE (hehp@gig.ac.cn)

First author: Hongping HE (hehp@gig.ac.cn)

SUMMARY

Total number of pages: 19 (1−19)

Total number of text: 3

Total number of tables: 3

Total number of figures: 8

Text S1 The discussion on the Mössbauer characterization results of natural magnetite samples.

Text S2 The digestion procedure of magnetite samples before ICP-AES analysis.

Text S3 The procedure of surface site density analysis.

Table S1 Electron probe microanalysis results for HN, DM and PZH magnetites.

Table S2 Mössbauer parameters for the natural magnetite samples.

Table S3 Fitted equations and kinetics constants for the generation of $^{\bullet}OH$ catalyzed by TS magnetite.

Figure S1 Map showing sampling sites of natural magnetites in China. The sampling map was generated by ArcGIS 9.3 software (ESRI, Redlands, California, USA).

Figure S2 Energy dispersive (EDS) spectra of TS and ZK magnetites.

Figure S3 Normalized Fe K-edge XANES spectra (a) and EXAFS spectra (b) for the natural magnetite samples, FeO, Fe_3O_4, and $FeTiO_3$.

Figure S4 Normalized Ti K-edge XANES spectra (a) and EXAFS spectra (b) for PZH and DM magnetite samples, ilmenite, titanomagnetite, anatase, and rutile.

Figure S5 Room temperature Mössbauer spectra of TS, ZK, HN, DM and PZH natural magnetite samples.

Figure S6 Effect of catalyst dosage (a) and H_2O_2 concentration (b) on the kinetics of $^{\bullet}OH$ generation at neutral pH. (a): 10 mmol L^{-1} of H_2O_2; (b): 1.0 g L^{-1} of magnetite.

Figure S7 UV-Vis spectra of p-NP during its degradation by TS magnetite.

Figure S8 The *pH-C*(H_s) curves (a) and *Cran*-V curves (b) for natural magnetites obtained from the acid-base potentiometric titration.

Text S1 The discussion on the Mössbauer characterization results of natural magnetite samples.

The Mössbauer spectral parameters of the TS and ZK magnetite samples indicate the sole phase of magnetite with hyperfine field (B_{hf}) of ~49.1±0.1 T (tetrahedral site A) and 46.0±0.3 T (octahedral site B) and isomer shifts (IS) of 0.29±0.1 and 0.66±0.1 mm s^{-1}, respectively (Figure S5 and Table S2). The relative ratios of the A to B site area is almost 0.5. The Mössbauer spectral parameters of TS and ZK magnetites are similar to those of pure magnetite[1]. This suggests that most of the trace metal cations do not enter the structure of magnetite, which is consistent with the EDS analysis results. For HN sample, its Mössbauer parameter is identical to that of maghemite (γ-Fe_2O_3), with B_{hf} of ~51.9 T and IS of 0.329 mm s^{-1}, respectively (Table S2). From ICP-AES measurements (Table 3) and Electron probe microanalysis (Table S1), the matrix of HN is magnetite, suggesting the oxidation of Fe^{2+} to Fe^{3+} without changing the magnetite structure[2,3], during its formation in the subduction-related environment with high oxygen fugacity[4]. Magnetite and maghemite cannot be distinguished by XRD. The disappearance of ilmenite on the Mössbauer spectrum of HN is probably ascribed to its low content. For the Mössbauer spectra of DM and PZH samples, the main signals is related to magnetite, with B_{hf} of ~49.0±0.1 T (tetrahedral site A) and 46.0±0.5 T (octahedral site B) and isomer shifts (IS) of 0.30±0.5 and 0.68±0.3 mm s^{-1}, respectively (Table S2). But for DM, the relative ratio of the A to B site area is about 0.4, indicating that the trace metal cations preferentially occupy the tetrahedral sites. For PZH, the relative ratio of the A to B site area is almost 0.5, indicating that trace metal cations do not enter the structure of

magnetite. Besides the sextet signals of magnetite on the spectra of DM and PZH, two doublet spectra appear. One doublet with IS of 1.0±0.1 mm s^{-1} is related to the ilmenite. The other doublet on DM spectrum with high QS of 2.58 mm s^{-1} and IS of 1.262 mm s^{-1} is probably related to the Fe^{2+} on the octahedral sites of pleonaste, while the doublet on PZH spectrum with high QS of 2.58 mm s^{-1} and IS of 0.663 mm s^{-1} is related to the Fe^{2+} and Fe^{3+} on the octahedral sites of pleonaste. The relative area of ilmenite in DM is 19.9%, obviously higher than that in PZH (8.2%), while the relative area of pleonaste in DM is 3.7%, lower than that in PZH (5.4%), indicating the higher ilmenite content and lower pleonaste content in DM than that in PZH, which is consistent to the XRD analysis results.

Text S2 The digestion procedure of magnetite samples before ICP-AES analysis.

1.0 g of sample was mixed with by 0.6 mL of HNO_3 (50% v/v) and 0.6 mL of HF in a digestion vessel. The vessel was tightly sealed and heated at 100 ^{o}C for 3 days to evaporate the solvent. The obtained residue was digested by repeating the above procedure. Then the residue was completely dissolved by 2.4 mL HCl, and then diluted 2000 times with Milli-Q water for ICP-AES analysis.

Text S3 The procedure of surface site density analysis.

The surface site density (D_s) of magnetite particles, i.e., the number of proton-active sites (per nm^2), was determined by acid-base potentiometric titration[5], by applying Eq.(1) :

$$D_s = (H_s \times N_A)/SC_s \times 10^{18} \quad (1)$$

where N_A is Avogadro's constant (6.02×10^{23}), S is the BET surface area ($m^2\ g^{-1}$), C_s is the concentration of magnetite particles ($1.0\ g\ L^{-1}$), and H_s is H^+ adsorption capacity ($mol\ L^{-1}$). H_s can be derived from Eq. (2):

$$H_s = V_e \times C_{alkali} \quad (2)$$

where V_e is the titration volume of alkali at the equivalence point, given by the intersection of the Gran titration curve and the X-axis[6], and C_{alkali} is the concentration of alkali used in titration. In addition, the intrinsic acidity constants of the surface (pK_{a1} and pK_{a2}), and the zero point of charge (pH_{zpc}), were obtained from the Gran titration curves (Figure S8).

In the titration, the sample was mixed with NaCl solutions of different concentrations (c) to obtain $1\ g\ L^{-1}$ magnetite suspensions in which the final concentrations of NaCl are 0.001, 0.010, and 0.100 $mol\ L^{-1}$. When the change in pH value did not exceed 0.02 pH unit in 5 min, the amount of acid or base added and the potential value of the suspension were recorded and used to calculate the adsorption amounts of H^+ and OH^-. Finally, potentiometric titration curves were obtained by plotting (OH^- and H^+) versus pH for different c values.

Table S1 Electron probe microanalysis results for HN, DM and PZH magnetites.

Samples	HN			DM			PZH	
Major oxides (*wt%*)	Magnetite host	Ilmenite	Pleonaste	Magnetite host	Ilmenite	Pleonaste	Magnetite and ilmenite host	Pleonaste and ilmenite
SiO_2	0.08	0.34	0.08	0.05	0.00	0.14	0.04	0.06
MgO	0.16	1.32	10.55	0.06	0.50	4.82	3.46	19.01
Al_2O_3	0.87	0.07	62.41	0.58	0.01	59.37	5.41	60.07
FeO	33.54	46.04	26.91	31.91	46.53	33.91	43.45	17.26
Fe_2O_3	62.26	0.24	0.00	66.17	0.72	0.00	30.65	0.00
MnO	0.13	0.86	0.26	0.00	0.80	0.22	0.37	0.09
Cr_2O_3	0.10	0.02	0.06	0.43	0.04	0.37	0.01	0.00
V_2O_3	1.15	0.11	0.18	0.80	0.05	0.10	0.52	0.14
TiO_2	2.30	50.72	0.34	0.57	51.73	0.09	14.17	3.02
Total	100.58	99.72	100.83	100.57	100.38	99.15	98.09	99.69

The matrix of HN and DM is composed of Fe and O, and contains low amounts of trace metals. The Fe and Ti content of exsolved ilmenite in HN and DM is close to the classical value for ilmenite, while the chemical composition (e.g., Fe, Al, Mg) of exsolved pleonaste is similar to that of pleonaste. For PZH, the magnetite and ilmenite matrix contains mainly Fe, O and Ti, while the exsolved phase of pleonaste contains high amounts of Al, Mg and Fe, and low amounts of Ti.

Table S2 Mössbauer parameters for the natural magnetite samples.

sample	phase	IS/mm s^{-1} (±0.002)	QS /mm s^{-1} (±0.004)	B_{hf} /T (±0.004)	Relative area (%)
TS	Magnetite(tet)	0.293	0.0100	49.160	33.4
	Magnetite(oct)	0.664	-0.00170	46.000	66.6
ZK	Magnetite(tet)	0.298	0.0236	49.153	33.6
	Magnetite(oct)	0.668	0.0276	45.794	66.4
HN	Maghemite	0.329	-0.182	51.892	100
DM	Magnetite(tet)	0.259	-0.0215	48.572	22.3
	Magnetite(oct)	0.709	0.0282	46.092	54.1
	Ilmenite	1.091	0.695	---	19.9
	pleonaste	1.262	2.58	---	3.7
PZH	Magnetite(tet)	0.342	-0.0175	49.098	28.8
	Magnetite(oct)	0.679	0.00780	45.533	57.6
	Ilmenite	1.063	-0.691	---	8.2
	pleonaste	0.663	2.00	---	5.4

Table S3 Fitted equations and kinetics constants for the generation of $^{\bullet}OH$ catalyzed by TS magnetite.

Sample	Fitted equation	R^2
TS-Blank	y = -1.56 + 0.010x	0.999
TS-0.25 g L^{-1}	y = 6.67 + 0.10x	0.982
TS-0.75 g L^{-1}	y = 20.6 + 0.11x	0.987
TS-1.0 g L^{-1}	y = 11.2 + 0.24x	0.996
TS-1.5 g L^{-1}	y = 36.6+ 0.29x	0.967
H_2O_2-Blank	y = 0.876 + 0.0054x	0.977
H_2O_2-5 mmol L^{-1}	y = 21.3 + 0.17x	0.993
H_2O_2-10 mmol L^{-1}	y = 11.2 + 0.24x	0.996
H_2O_2-20 mmol L^{-1}	$y = 61.6 + 0.0017x^{1.70}$	0.998
H_2O_2-40 mmol L^{-1}	$y = 73.6 + 0.033x^{1.34}$	0.997
H_2O_2-60 mmol L^{-1}	$y = 206.2 + 7.83\times10^{-5}x^{2.24}$	0.992

The rate of $^{\bullet}OH$ generation increased gradually as the magnetite dosage was increased from 0 to 1.5 g L^{-1}. The $^{\bullet}OH$ generation rate followed zero-order kinetics (linear regression). The rate of $^{\bullet}OH$ generation also increased with a rise in H_2O_2 concentration. When the H_2O_2 concentration increased from 5 to 60 mmol L^{-1}, the kinetics changed from zero-order to a power function.

Figures

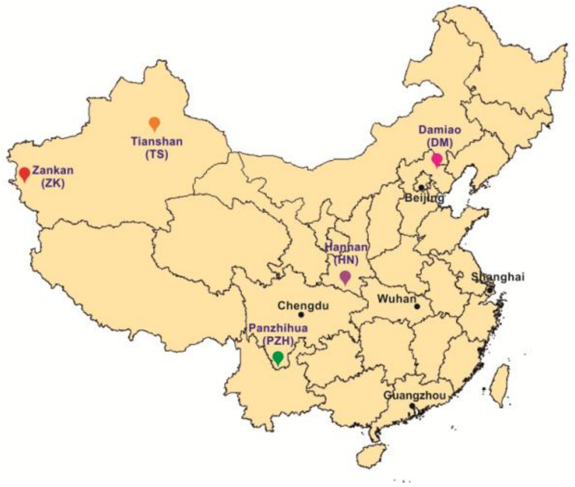

Figure S1 Map showing sampling sites of natural magnetites in China. The sampling map was generated by ArcGIS 9.3 software (ESRI, Redlands, California, USA). Purple pointer: Bijigou Fe-Ti oxide-bearing, mafic-ultramafic intrusion in the Hannan complex, central China (HN); Green pointer: Panzhihua Fe-Ti oxide-bearing layered intrusion, SW China (PZH); Pink pointer: Damiao Fe-Ti-P oxide-bearing anorthosite massif, north China (DM); Orange pointer: Dahalajunshan iron oxide deposit, south Tianshan (TS); Red pointer: Zankan banded iron formation, NW China (ZK).

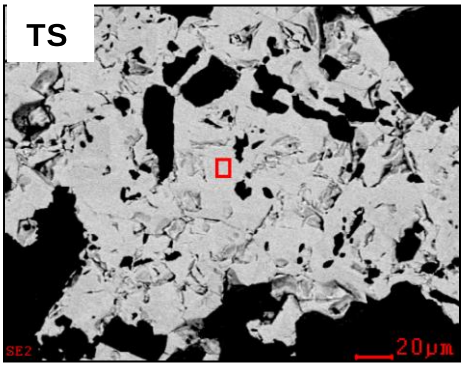

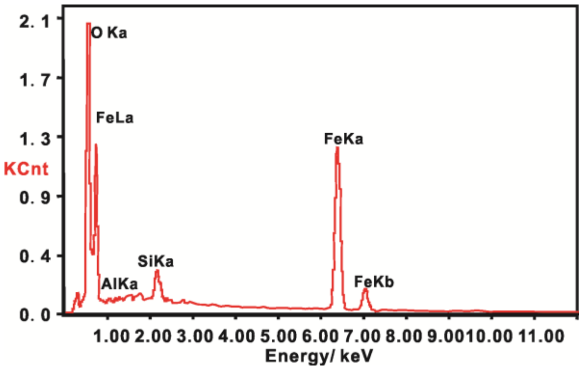

Element	*Wt%*	*At%*
OK	19.99	46.35
AlK	00.19	00.27
SiK	00.41	00.54
CaK	00.33	00.31
FeK	79.08	52.54

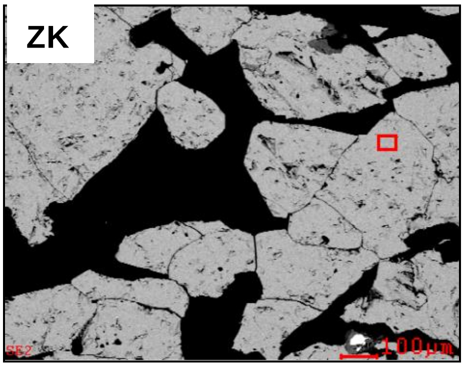

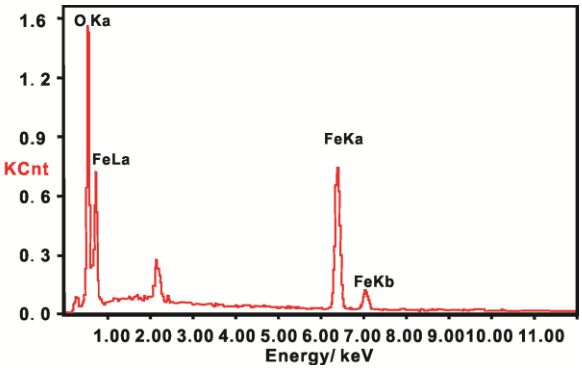

Element	*Wt%*	*At%*
OK	18.23	43.77
FeK	81.77	56.23

Figure S2 Energy dispersive (EDS) spectra of TS and ZK magnetites. The magnetite host of TS and ZK samples is composed of Fe and O, but ZK sample is with quite low contents of trace elements (e.g., Al, Si, Ca).

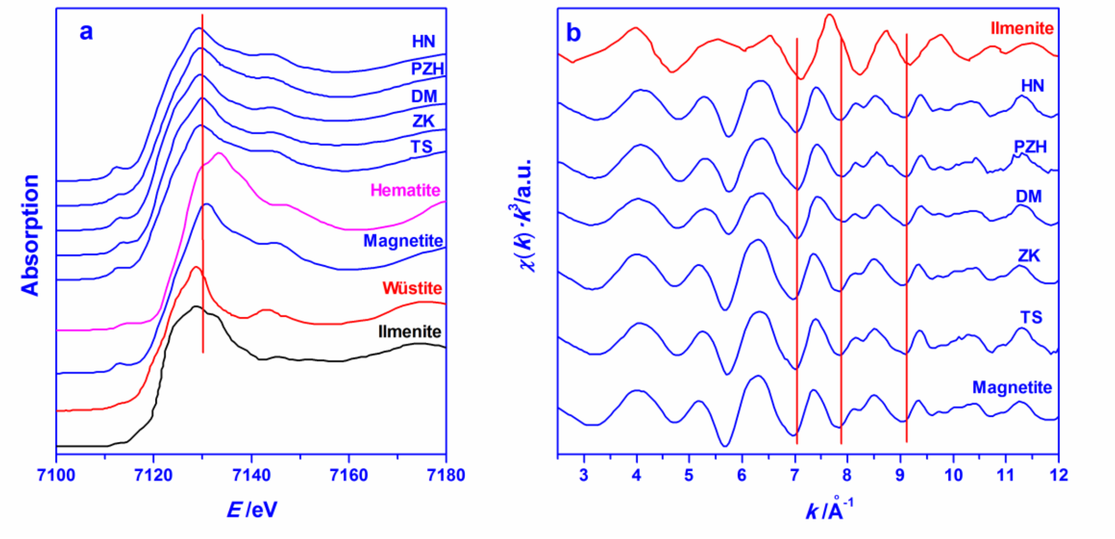

Figure S3 Normalized Fe K-edge XANES spectra (a) and EXAFS spectra (b) for the natural magnetite samples, FeO, Fe_3O_4, and $FeTiO_3$. The absorption edge positions of Fe species in TS, ZK, DM, HN and PZH are clearly different from the position of Fe^{3+} in Fe_2O_3, but close to that in synthetic magnetite. For DM, HN and PZH, they are even more closed to that of Fe^{2+} in FeO and ilmenite. The filtered EXAFS oscillations, $k^3\chi(k)$, of TS and ZK are almost identical with those of the synthetic mineral, but clearly different from those of ilmenite. For DM, HN and PZH, the oscillations display slight shift to high k value, especially in range of 7-9.5 $Å^{-1}$, and their positions become close to those of ilmenite.

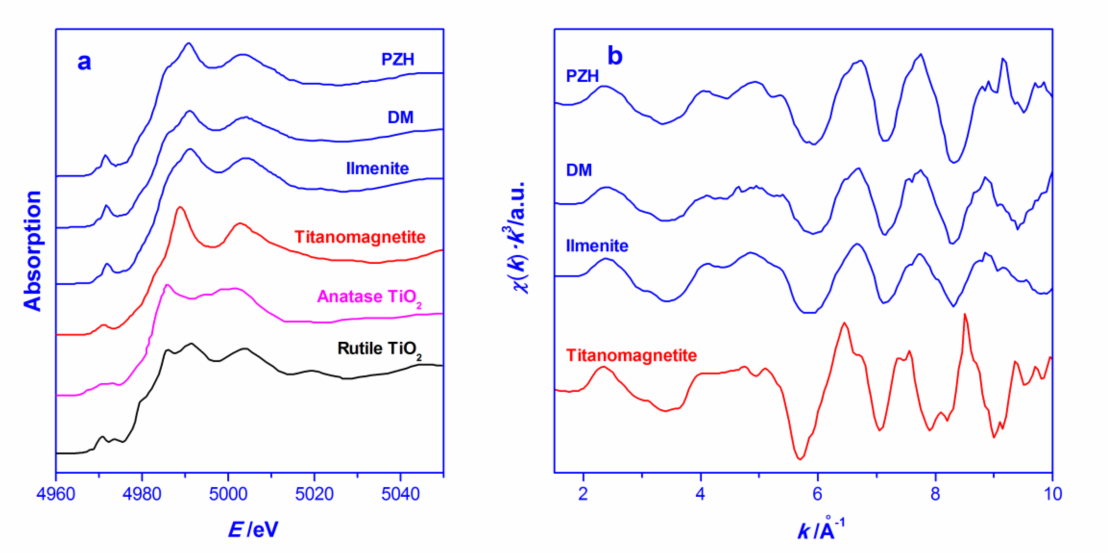

Figure S4 Normalized Ti K-edge XANES spectra (a) and EXAFS spectra (b) for PZH and DM magnetite samples, ilmenite, titanomagnetite, anatase, and rutile. The absorption position of Ti in and DM and PZH is close to that of Ti^{4+} in anatase, rutile, ilmenite, and titanomagnetite. The oscillations for natural magnetites are similar to that for ilmenite, but clearly different from those for titanomagnetite.

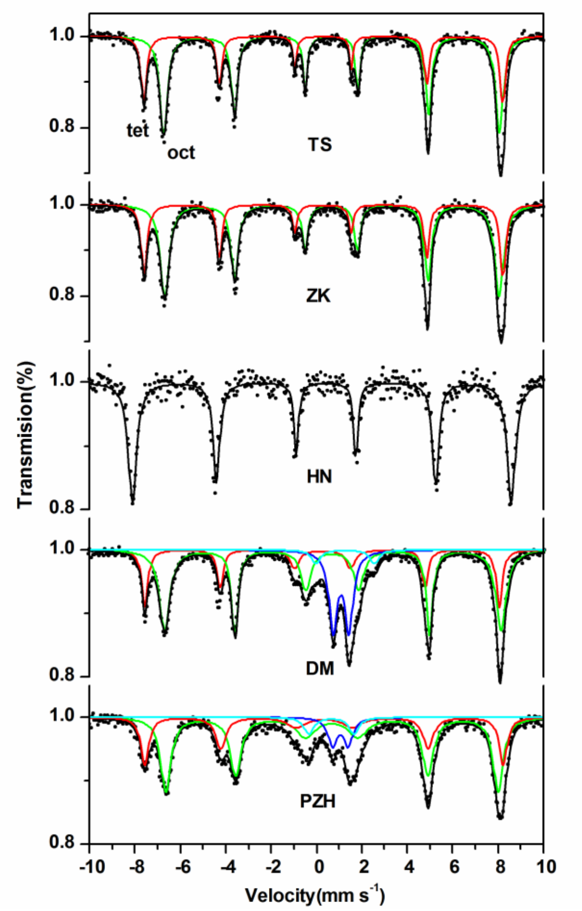

Figure S5 Room temperature Mössbauer spectra of TS, ZK, HN, DM and PZH natural samples.

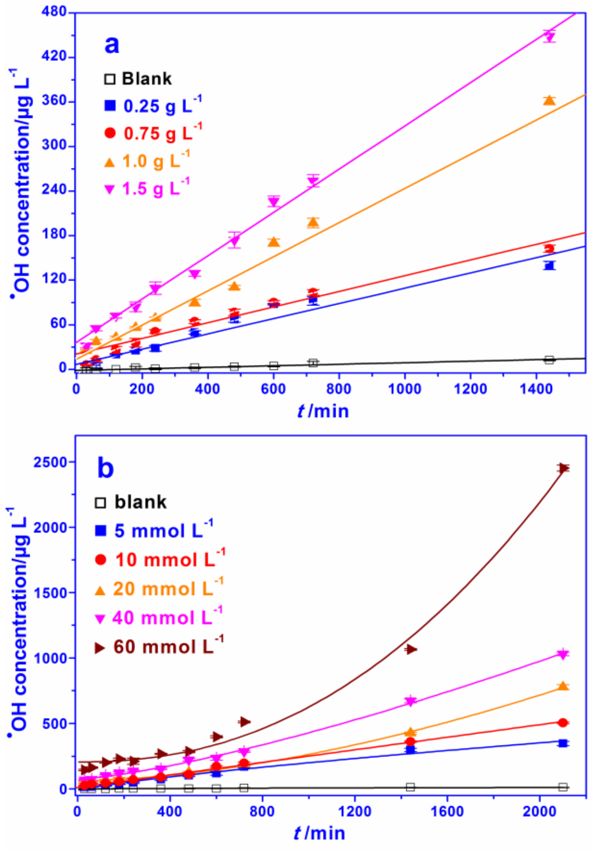

Figure S6 Effect of catalyst dosage (a) and H_2O_2 concentration (b) on the kinetics of $^{\bullet}OH$ generation at neutral pH. (a): 10 mmol L^{-1} of H_2O_2; (b): 1.0 g L^{-1} of magnetite. A gradual increase in the yield of $^{\bullet}OH$ was detected when the magnetite dosage increased from 0 to 1.5 g L^{-1}. The processes followed zero-order kinetics. The $^{\bullet}OH$ production also increased with H_2O_2 concentration. Up to a concentration of 10 mmol L^{-1}, $^{\bullet}OH$ production increased linearly with the reaction time, but when the peroxide concentration was further increased, the rate of $^{\bullet}OH$ generation changed from being linear to a power function.

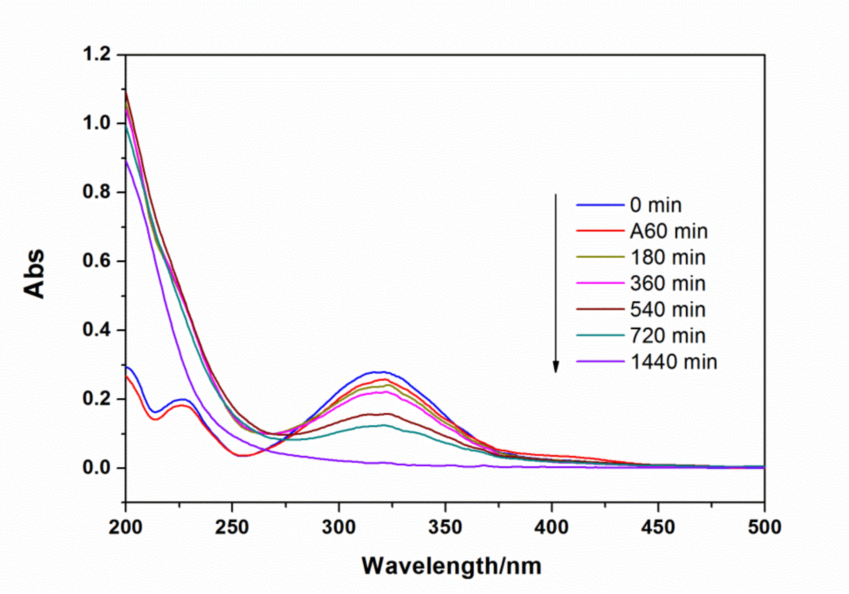

Figure S7 UV-Vis spectra of p-NP during its degradation by TS magnetite. Before addition of H_2O_2, the spectrum (0 min) showed two peaks at ~ 227 and 317 nm, corresponding to the phenyl ring in p-NP, and the π-conjugation between the phenyl ring and nitro group in p-NP, respectively. After addition of H_2O_2, a new peak appeared at ~ 200 nm due to H_2O_2. The peak at ~ 317 nm decreased with time of contact, indicating progressive degradation of p-NP.

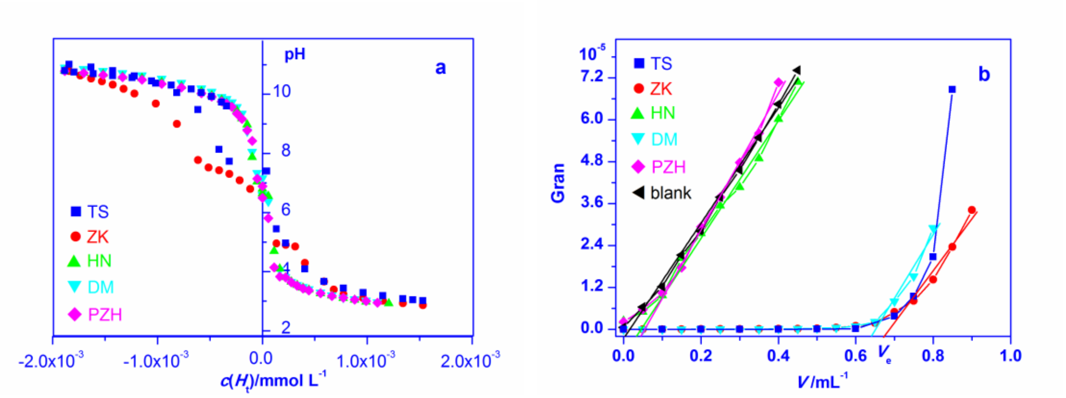

Figure S8 The *pH*-*C*(H_s) curves (a) and *Cran*-V curves (b) for natural magnetites obtained from the acid-base potentiometric titration. From the acid-base titration curves, the intrinsic acidity constants of magnetite surface (pK_{a1} and pK_{a2}), and the zero point of charge (pH_{zpc}) were from Figure S8.a. The surface site density (D_s) was obtained from Eqs. (1)-(2) and Figure S8.b.

References

1 Li, Y. L., Zhu, S. Y., & Deng, K. Mossbauer hyperfine parameters of iron species in the course of Geobacter-mediated magnetite mineralization. *Phys. Chem. Miner.* **38**, 701-708 (2011).

2 Magalhaes, F. *et al.* Cr-containing magnetites $Fe_{3-x}Cr_xO_4$: The role of Cr^{3+} and Fe^{2+} on the stability and reactivity towards H_2O_2 reactions. *Appl. Catal. A-gen.* **332**, 115-123 (2007).

3 Shukoor, M. I. *et al.* Multifunctional polymer-derivatized gamma-Fe_2O_3 nanocrystals as a methodology for the biomagnetic separation of recombinant His-tagged proteins. *J. Magn. Magn. Mater.* **320**, 2339-2344 (2008).

4 Zhao, J. H., Zhou, M. F., Yan, D. P., Zheng, J. P., & Li, J. W. Reappraisal of the ages of Neoproterozoic strata in South China: No connection with the Grenvillian orogeny. *Geology* **39**, 299-302 (2011).

5 Madden, A. S. & Hochella, M. F. A test of geochemical reactivity as a function of mineral size: Manganese oxidation promoted by hematite nanoparticles. *Geochim. Cosmochim. Acta* **69**, 389-398 (2005).

6 Hu, W. G., Su, Y. L., Sun, D. J., & Zhang, C. G. Studies on zero point of charge and permanent charge density of Mg-Fe hydrotalcite-like compounds. *Langmuir* **17**, 1885-1888 (2001).
